# The role of synbiotics as adjunctive agents in the treatment of allergic rhinitis: A randomized controlled trial

**DOI:** 10.1002/hsr2.1571

**Published:** 2023-10-03

**Authors:** Arezoo Faridzadeh, Yaser Yadegari, Mahdi Bakhshaee, Mona Kabiri, Mozhgan Mohammadi, Maryam Khoshkhui, Farahzad Jabbari Azad

**Affiliations:** ^1^ Immunology Research Center Mashhad University of Medical Sciences Mashhad Iran; ^2^ Department of Immunology and Allergy, School of Medicine Mashhad University of Medical Sciences Mashhad Iran; ^3^ Allergy Research Center Mashhad University of Medical Sciences Mashhad Iran; ^4^ Department of Otolaryngology, Qaem Hospital Mashhad University of Medical Sciences Mashhad Iran; ^5^ Nanotechnology Research Center, Pharmaceutical Technology Institute Mashhad University of Medical Sciences Mashhad Iran; ^6^ Clinical Research Development Unit, Ghaem Hospital Mashhad University of Medical Sciences Mashhad Iran

**Keywords:** allergic rhinitis, immunoglobulin E, prebiotic, probiotic, quality of life, synbiotics

## Abstract

**Introduction:**

Allergic rhinitis (AR) is a prevalent chronic disease affecting a significant portion of the global population. The substantial economic burden associated with treating AR necessitates the exploration of alternative therapies. Probiotics have gained attention due to their availability, minimal adverse effects, and cost‐effectiveness. The present study aims to investigate the role of synbiotics as adjunctive agents in the treatment of AR when added to standard treatment.

**Method:**

Thirty patients with persistent allergic rhinitis (PAR) were randomly assigned to receive routine diet therapy plus synbiotics or routine diet therapy plus placebo per day for 4 months. The data analysis was conducted using SPSS Version 20.

**Result:**

This study revealed a notable difference in immunoglobulin (Ig)E levels between the placebo and synbiotics groups (*p* = 0.035) following the intervention. Although a statistically significant difference (*p* = 0.039) was observed in the changes before and after the intervention (synbiotics and placebo) in the SNOT22 questionnaire, this finding was not observed for the MiniRQLQ questionnaire. For the MiniRQLQ questionnaire, the within‐group analysis showed significant changes in activity variables (*p* = 0.023), ocular symptoms (*p* = 0.036), and practical problems (*p* = 0.043) exclusively in the synbiotics group. Additionally, changes in nasal symptoms were observed in both synbiotics (*p* = 0.006) and placebo (*p* = 0.007) groups.

**Conclusion:**

This study suggests that synbiotics supplementation for 4 months can impact IgE levels compared with placebo in individuals with PAR, while also exhibiting positive effects on symptomology.

## 1 INTRODUCTION

Allergic rhinitis (AR) is a prevalent chronic disease worldwide and has been estimated to affect about 10%–40% of the general population.^1^ Billions of dollars are spent each year on treating AR, which places a huge burden on the healthcare system in the United States.^2^

It is subdivided into seasonal (SAR) and perennial (PAR) diseases based on the duration of allergen exposure. AR is a type I hypersensitivity (immunoglobulin E [IgE]‐mediated disease) caused by the degranulation of mast cells.^3^

AR is characterized by rhinorrhea, nasal itching, sneezing, insomnia, sleep disturbances, learning difficulties, and decreased concentration and productivity in school activities, all of which affect the quality of life (QOL).^4^

The main treatments for allergies are avoidance of allergens, antihistamines, corticosteroids, and bronchodilators, which are effective but may have adverse effects (e.g., sedation, nasal irritation, epistaxis, increased blood pressure, dizziness, headache).^5^

Therefore, trying to find ways to choose complementary treatments is still one of the topics of interest. Probiotics, which have received considerable scientific attention over the past few decades, might be an alternative therapeutic modality since of their easy availability, minimal adverse events, and relatively low cost.^6^

Probiotics are nonpathogenic commensal microorganisms that when administered in sufficient amounts, benefit the normal state of the host's intestine. They can modulate the mucosal immune system and prevent an inflammatory condition of allergy and atopy.^7^, ^8^, ^9^, ^10^, ^11^, ^12^, ^13^, ^14^ Prebiotics are nutritional elements that selectively stimulate the activity and growth of colon‐residing bacteria, comprising indigestible carbohydrates such as galactooligosaccharides, fructooligosaccharides (FOS), inulin, and lactose. The mixture of probiotics and prebiotics is defined as synbiotics.^13^, ^15^

A recently conducted randomized clinical trial investigated the effect of synbiotics in allergic patients. However, the study had a smaller sample size, and the evaluation of IgE levels was not included.^16^

The present study aimed to investigate the effectiveness of synbiotics in combination with standard treatment in patients with PAR who were sensitive to *Salsola Kali*, one of the most common allergens in the northeastern region of Iran. The primary outcome was the effects on IgE level, the secondary outcome was the effect on QoL and AR symptoms.

## 2 METHOD

### 2.1 Study design

The study was conducted as a double‐blind, placebo‐controlled, and randomized clinical trial. The protocol was approved by the Ethics Committee of Mashhad University of Medical Sciences (IR. MUMS.MEDICAL REC.1397.477), and the registration number ID in the Iranian Registry of Clinical Trail (IRCT) was IRCT20150716023235N14.

### 2.2 Characterization of patients

The study was conducted on 30 patients aged between 5 and 50 years, suffering from persistent allergic rhinitis sensitized to *Salsola Kali* (positive prick test). The study was carried out from 2020 to 2021 at the allergy clinic of Qaim Hospital in Mashhad, Iran. Patients were included if they had moderate‐to‐severe PAR over the previous 2 years and did not respond adequately to medical treatment, including corticosteroid nasal spray, nasal decongestants, and antihistamines. All participants provided written consent. Exclusion criteria included pregnancy, lactation, history of antibiotic and multivitamin/mineral supplements intake during enrollment, infections, malnutrition, or allergic asthma, immunodeficiency, autoimmune disorders, systemic corticosteroid or immunosuppressive medicine usage, and probiotic intake in the last 3 months. Due to the quarantine period of COVID‐19, the patients did not exercise regularly, and none reported smoking and hookah consumption. Patients maintained a typical diet comprising four food groups during the study.

### 2.3 Randomization and study groups

To ensure the randomization, a quadruple‐block randomization approach was used, along with randomized blind tables. Patients were randomly assigned to the placebo or synbiotics group. Clinical assessors, analysts, and patients were kept unaware of the group assignments, thus maintaining blinding.

The synbiotics group included 15 patients with PAR who received routine diet therapy (intranasal steroid and antileukotriene or antihistamine) as along with synbiotics capsules from “ZistTakhmir” company (www.zisttakhmir.com). The capsules contained 10^9^ CFU probiotics, including *Lactobacillus (L). bulgaricus*, *L. acidophilus, L. rhamnosus*, *L. casei*, *Bifidobacterium (B). longum*, *B. breve*, *Streptococcus thermophilus*, and FOS (fructooligosaccharide) as a prebiotic. Another 15 patients underwent routine diet therapy and received a visually identical placebo. Both groups took one capsule daily after lunch for 4 months. The placebo was identical in appearance to the actual synbiotics provided by the company. Patients did not consume any other probiotic products during the intervention.

### 2.4 Outcome

The primary outcome was *Salsola Kali*‐specific IgE levels, measured through peripheral blood samples collected before and after the intervention. The RAST (Radioallergosorbent test) method quantified specific IgE antibodies targeting the *Salsola Kali* allergen.

The secondary outcome assessed clinical symptoms and QOL using the standard SNOT‐22 questionnaire and the mini‐Rhinoconjunctivitis Quality of Life Questionnaire (miniRQLQ) at 0 and 4 months, respectively. The miniRQLQ was used to assess different domains of symptoms, including activity, functional problems, nasal symptoms, ocular symptoms, and others.

### 2.5 Statistical analysis

Data were collected and analyzed using SPSS software version 20. The Kolmogorov–Smirnov test checked normal distribution of quantitative variables. Mean ± standard deviation (SD) expressed normally distributed data, while median (IQR) expressed nonnormal variables. The intention‐to‐treatment method (ITT) was used. Mann–Whitney test compared nonnormal variables, and independent sample *T* test compared normal variables between groups. Within‐group comparisons used Wilcoxon signed‐rank test or paired sample *T* test. Qualitative variables were described by frequency (percentage) and analyzed using *χ* ^2^ or Fisher's exact test. A *p* value < 0.05 was considered statistically significant.

## 3 RESULTS

### 3.1 Participants

**Figure 1:**
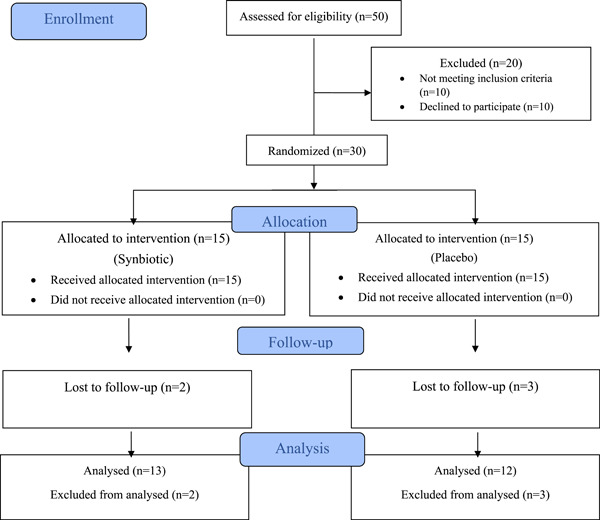
Study CONSORT diagram.

Thirty patients with PAR were randomly assigned to receive routine diet therapy plus synbiotics (*n* = 15) or routine diet therapy plus Placebo (*n* = 15). Five patients (two in the synbiotics group and three in the placebo group) discontinued treatment during the study. One patient was excluded due to gastrointestinal complications like diarrhea and flatulence, two patients due to secondary infections requiring antibiotics, and two due to lack of family consent. At the end of the study, three patients in the synbiotics group and two patients in the placebo group were unable to provide final serum IgE measurements due to reasons such as long distance, fear of COVID‐19 infection, and other factors (Figure 1).

### 3.2 Demographics

The study findings indicate no significant differences in age between the synbiotics and placebo groups, with mean ages of 26.15 years (SD = 10.27) and 26.92 years (SD = 13.75), respectively (*p* = 0.473). The gender distribution analysis also showed no statistically significant difference between the synbiotics and placebo groups. The synbiotics group comprised 4 male participants (26.7%) and 11 female participants (73.3%), while the placebo group included 9 male participants (60%) and 6 female participants (40%) (*p* = 0.139).

### 3.3 IgE levels

**Figure 2:**
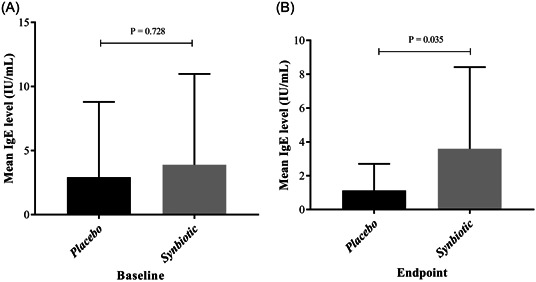
Changes in IgE levels before and after intervention in the placebo and synbiotics groups. (A) Comparison of changes between the placebo and synbiotics groups before the intervention revealed no statistical significance (*p* = 0.728). (B) Significant differences in changes after the intervention were observed between the placebo and synbiotics groups (*p* = 0.035).

Following the intervention, a significant difference in IgE levels was observed between the placebo and synbiotics groups (*p* = 0.035). However, there was no significant difference in IgE levels before and after the intervention within either the placebo or synbiotics groups (Table 1, Figure 2).

**Table 1:** Effect of intervention on the quality of life, clinical symptoms, and serum IgE levels of patients with PAR in the synbiotics and placebo groups at pre‐ and posttreatment.

Variables^a^	Placebo group			Synbiotics group			P1	P2	P3	P4	P5
	Baseline	Endpoint	Change	Baseline	Endpoint	Change					
*IgE level, (UI/mL)*							0.728	**0.035**	0.203	0.333	0.853
Mean ± SD	2.92 ± 5.88	1.12 ± 1.58	−0.24 ± 0.6	3.88 ± 7.09	3.59 ± 4.82	−1.02 ± 2.95					
Median (IQR)	0.9 (0.26, 1.7)	0.55 (0.26, 1.64)	−0.21 (−0.82, 0.07)	3.4 (0.44, 3.7)	2.28 (1.27, 2.87)	−0.22 (−1.27, 0.76)					
*MiniRQLQ*							0.757	0.087	**0.021**	**0.013**	0.248
Mean ± SD	26.16 ± 12.91	11.3 ± 15.94	−10.66 ± 13.28	30.30 ± 18.54	15.5 ± 11.45	−19 ± 21.13					
Median (IQR)	25 (15, 38)	14.5 (5.5, 23)	−5.5 (−20, −0.5)	27 (20, 42)	5 (1, 11)	−13 (−33, −6)					
*SNOT22*							0.260	0.406	**0.011**	**0.001**	**0.039**
Mean ± SD	33.08 ± 19.93	20.33 ± 16.74	−12.75 ± 15.7	44.38 ± 19.17	18.38 ± 21.29	−26 ± 14.35					
Median (IQR)	25 (16.5, 51)	15.5 (11, 22)	−9 (−20, −3.5)	46 (37, 52)	11 (3, 19)	−23 (−41, −14)					

*Note*: P1, Between groups at baseline. Significant *p* values are bolded.

P2, Between groups at the endpoint.

P3, Within the placebo group (compare baseline and endpoint of follow up in the placebo group).

P4, Within the synbiotic group (compare baseline and endpoint of follow up in the synbiotic group).

P5, Between groups based on Change.

Abbreviations: IgE, immunoglobulin E; IQR, interquartile range; MiniRQLQ, Mini Rhinoconjunctivitis Quality of Life Questionnaire; SD, standard deviation; SNOT22, The 22‐item Sinonasal Outcome Test.

^a^ Nonnormal distribution.

### 3.4 Questionnaire results

Table 1 presents that in both the synbiotics and placebo groups, as well as in both questionnaires, the mean scores after the intervention decreased compared with before. Although a statistically significant difference (*p* = 0.039) was observed in the changes before and after the intervention (synbiotics and placebo) in the SNOT22 questionnaire, this finding was not observed for the MiniRQLQ questionnaire.

In the intragroup analysis, both the synbiotics and placebo groups exhibited a significant difference in the mean scores of the questionnaires before and after the intervention. This suggests notable improvements in the scores after the intervention compared with before, for both the synbiotics and placebo groups. However, the level of significance was greater for both questionnaires in the synbiotics group.

As shown in Table 2, the miniRQLQ questionnaire consists of five domains: activity, ocular symptoms, nasal symptoms, practical problems, and other symptoms. The results demonstrate a statistically significant difference in nasal symptoms between the groups after the intervention (*p* = 0.04). Additionally, a significant difference was observed in other symptoms regarding the change before and after the intervention (*p* = 0.04).

**Table 2:** Comparative analysis of MiniRQLQ domains in patients receiving placebo, or synbiotics.

Variables^a^	Placebo group			Synbiotics group			P1	P2	P3	P4	P5
	Baseline	Endpoint	Change	Baseline	Endpoint	Change					
*Activity*							0.629	0.870	0.064	**0.023**	0.392
Mean ± SD	3.91 ± 4.18	2 ± 2.62	−1.91 ± 3.36	5.76 ± 4.72	2.53 ± 2.87	−3.23 ± 4.14					
Median (IQR)	2.5 (0.5, 7)	1.5 (0, 3)	−1 (−2.5, 0)	8 (1, 9)	2 (0, 4)	−3 (−6, 0)					
*Practical problems*							0.673	0.270	0.050	**0.043**	0.769
Mean ± SD	4.83 ± 3.45	2 ± 2.25	−2.83 ± 3.99	4.69 ± 4.34	1.53 ± 2.81	−3.15 ± 4.74					
Median (IQR)	6 (1, 8)	1.5 (0.5, 2.5)	−2 (−5.5, 0.5)	4 (0, 8)	2 (0, 2)	−1 (−8, 0)					
*Nasal symptoms*							0.967	**0.040**	**0.007**	**0.006**	0.252
Mean ± SD	8.83 ± 3.95	4.83 ± 4.17	−4 ± 3.95	8.69 ± 5	2.38 ± 4.03	−6.3 ± 5.73					
Median (IQR)	9.5 (6, 12)	4 (1.5, 8.8)	−4 (−6.5, −1.5)	9 (6, 13)	0 (0, 3)	−6 (−10, −2)					
*Ocular symptoms*							0.654	0.376	0.085	**0.036**	0.186
Mean ± SD	5 ± 4.72	2.58 ± 2.42	−2.41 ± 5.03	6.07 ± 5.28	2.15 ± 3.53	−3.92 ± 6.29					
Median (IQR)	4 (1.5, 7)	3 (0, 4)	−0.5 (−1.5, 0)	6 (2, 9)	0 (0, 3)	−3 (−6, 0)					
*Other*							0.497	0.205	0.391	0.075	**0.040**
Mean ± SD	3.58 ± 3.02	4.08 ± 3.44	0.5 ± 1.97	5.07 ± 5.23	2.69 ± 4.19	−2.38 ± 5.07					
Median (IQR)	3.5 (0.5, 6)	4 (0.5, 6)	0 (−0.5, 2)	3 (0, 9)	1 (0, 3)	−1 (−3, 0)					

*Note*: P1, Between groups at baseline. Significant *p* values are bolded.

P2, Between groups at the endpoint.

P3, Within the placebo group (compare baseline and endpoint of follow up in the placebo group).

P4, Within the synbiotics group (compare baseline and endpoint of follow up in the synbiotics group).

P5, Between groups based on Change.

Abbreviations: IQR, interquartile range; SD, standard deviation.

^a^ Nonnormal distribution.

Based on the within‐group analysis, notable variations were detected in the variables of activity (*p* = 0.023), ocular symptoms (*p* = 0.036), and practical problems (*p* = 0.043) exclusively within the synbiotics group. Moreover, changes in nasal symptoms were found in both the placebo (*p* = 0.007) and synbiotics (*p* = 0.006) groups.

## 4 DISCUSSION

The aim of this randomized, double‐blind, placebo‐controlled trial was to examine the effect of synbiotics supplementation on clinical symptomology and serum IgE levels in individuals with PAR for 4 months.

This study showed a significant difference in IgE levels between the placebo and synbiotics groups after the intervention. However, there was no significant change in IgE levels before and after the intervention in each group separately. These findings suggest that synbiotics supplementation may affect IgE levels compared with placebo in subjects with PAR.

Additionally, these findings suggest that the intervention had a positive impact on symptomology as measured by the SNOT22 questionnaire and MiniRQLQ questionnaire. However, the comparison of changes before and after the intervention between the two groups was found to be significant in the SNOT22 questionnaire, but not in the MiniRQLQ questionnaire.

The miniRQLQ questionnaire revealed a significant difference in nasal symptoms and other symptoms between the groups after the intervention. Significant variations were observed within the synbiotics group for activity, ocular symptoms, and practical problems, while changes in nasal symptoms were found in both the placebo and synbiotics groups.

Therefore, the findings are consistent with the conducted studies. A randomized, double‐blind, placebo‐controlled study conducted in children up to 2 years of age, who had allergic atopic dermatitis (AD) and cow's milk protein (CMP) allergy, demonstrated that the administration of a probiotic containing a blend of *L. rhamnosus* and *L. casei* strains was both safe and led to a significant improvement in the severity of AD symptoms.^17^

In an Iranian randomized clinical trial, the effect of synbiotics on patients with PAR was investigated over an 8‐week period, with participants assigned to either a synbiotics group or a placebo group. The results demonstrated a greater reduction in the SNOT‐22 and mini‐RQLQ scores in the synbiotics group, although the difference was not statistically significant.^18^ However, in our study, which spanning a 4‐month intervention period, we observed a significant reduction in the SNOT‐22 score. Additionally, we also observed a significant decrease in the level of IgE after the intervention in the synbiotics group.

An experimental study has also demonstrated the positive effects of probiotics. For instance, the colonization of germ‐free mice with a combination of *L. rhamnosus* and *L. casei* resulted in an enhancement of intestinal mucosa integrity and an improvement in allergic sensitivity.^19^ Similarly, oral feeding of *L. bulgaricus* attenuated allergic asthma‐induced inflammation and airway remodeling in a murine model of allergic asthma.^20^

In a randomized clinical trial with 49 patients suffering from PAR for 8 weeks, the oral administration of *L. acidophilus* was found to effectively reduce PAR symptoms. However, no statistically significant changes observed in the blood parameters during the study.^21^

In a small‐scale double‐blind, placebo‐controlled RCT conducted in Iran involving children and adults (*n* = 20, aged 9–53 years), the study examined the effectiveness of combining immunotherapy with a synbiotics treatment consisting of *Streptococcus thermophilus, L. bulgaricus, L. acidophilus, L. casei, L. rhamnosus, B. longum, B. breve*, and fructo‐oligosaccharide. The results revealed a significant lowering in IL‐17 gene expression after 2 and 6 months in the group receiving immunotherapy and the synbiotics, compared with the group obtaining immunotherapy and a placebo.^16^

Dennis Wall et al. demonstrated that the probiotic mixture (*L. gasseri*, *B. bifidum*, and *B. longum*) had a positive effect on seasonal sensitivity when compared with a placebo, as evidenced by the improvement in the global MRQLQ score from baseline to peak pollen.^22^

A recent meta‐analysis demonstrated the potential benefits of specific probiotic strains in improving symptoms of AR and enhancing QOL.^23^ According to the studies, it appears that the concurrent administration of multiple probiotics exerts a synergistic effect on the amelioration of allergic diseases,^16^, ^24^, ^25^ whereas, in certain studies, the administration of individual probiotic strains shows no impact on allergy improvement.

In this regard, John et al. reported that treatment with *L. rhamnosus* did not reduce rhinitis symptoms or immune parameters in symptomatic children.^26^

A double‐blind RCT investigating the impact of *L. paracasei* on 131 individuals with grass pollen allergy revealed no statistically significant differences in specific IgE levels, QOL, or AR symptom scores between the group receiving *L. paracasei* and the placebo group.^27^ Also, Helin et al. found no evidence indicating a beneficial therapeutic effect of *L. rhamnosus* in patients with atopic allergies.^28^

In a systematic review, a positive effect of probiotics in improving RQLQ and SNOT22 scores was observed, which is consistent with our study.^23^ However, this study noted that probiotics did not lead to significant changes in IgE levels. It is worth noting that both our clinical trial study and another RCT study^29^; with intervention periods of 16 and 13 weeks, respectively, demonstrated a significant decrease in IgE levels within the probiotic group. Furthermore, it appears that the administration of multiple probiotic strains together may have a more pronounced effect on immune system regulation due to their synergistic effect.^16^

## 5 LIMITATION

In summary, the main limitation of this study is the small number of enrolled patients. Consequently, it is recommended that larger‐scale multicenter trials involving a broader population of allergic patients would be beneficial.

## 6 CONCLUSION

In conclusion, the findings of this discussion suggest that synbiotics supplementation for 4 months may impact IgE levels compared with placebo in individuals with PAR. The intervention also showed positive effects on symptomology, as measured by the SNOT22 and MiniRQLQ questionnaires, with significant variations observed within the synbiotics group for activity, ocular symptoms, and practical problems. Other studies have highlighted the safety and efficacy of specific probiotic strains, such as *L. casei* and *L. rhamnosus* in improving AD symptoms, enhancing intestinal mucosa integrity, and reducing PAR symptoms. However, not all studies have demonstrated significant therapeutic effects of probiotics on allergies. Further research, including larger randomized controlled trials with specific probiotic strains, is needed to establish the efficacy of probiotic interventions in allergic diseases.

## AUTHOR CONTRIBUTIONS

**Arezoo Faridzadeh**: Data curation; investigation; methodology; supervision; validation; writing—original draft; writing—review and editing. **Yaser Yadegari**: Data curation; investigation; methodology; resources; validation; writing—original draft. **Mahdi Bakhshaee**: Data curation; investigation; resources; supervision; validation; visualization. **Mona Kabiri**: Conceptualization; investigation; methodology; resources; software; validation; writing—review and editing. **Mozhgan Mohammadi**: Conceptualization; investigation; methodology; resources; validation; visualization. **Maryam Khoshkhui**: Conceptualization; data curation; investigation; resources; validation. **Farahzad Jabbari Azad**: Conceptualization; funding acquisition; investigation; project administration; resources; supervision; writing—review and editing.

## CONFLICT OF INTEREST STATEMENT

The authors declare no conflict of interest.

## ETHICS STATEMENT

This study was approved by the Ethics Committee of Mashhad University of Medical Sciences (IR. MUMS.MEDICAL REC.1397.477).

## TRANSPARENCY STATEMENT

The lead author Farahzad Jabbari Azad affirms that this manuscript is an honest, accurate, and transparent account of the study being reported; that no important aspects of the study have been omitted; and that any discrepancies from the study as planned (and, if relevant, registered) have been explained.

## Data Availability

All authors have read and endorsed the definitive version of the manuscript. The corresponding author possessed comprehensive access to all data within this study and bears full responsibility for both the data's integrity and the accuracy of the data analysis. The data supporting the findings of this study are accessible through the corresponding author (Farahzad Jabbari Azad) upon submission of a reasonable request.
